# Marmosets treated with oxytocin are more socially attractive to their long-term mate

**DOI:** 10.3389/fnbeh.2015.00251

**Published:** 2015-10-14

**Authors:** Jon Cavanaugh, Michelle C. Huffman, April M. Harnisch, Jeffrey A. French

**Affiliations:** ^1^Callitrichid Research Center, Department of Psychology, University of Nebraska OmahaOmaha, NE, USA; ^2^Department of Biology, University of Nebraska OmahaOmaha, NE, USA

**Keywords:** Pro^8^-OXT, social relationships, affiliative behavior, pair-bond maintenance, proximity, grooming

## Abstract

Adult male-female bonds are partly characterized by initiating and maintaining close proximity with a social partner, as well as engaging in high levels of affiliative and sociosexual behavior. Oxytocin (OXT), a neuromodulatory nonapeptide, plays a critical role in the facilitation of social bonding and prosocial behavior toward a social partner (Feldman, 2012). However, less attention has been given to whether augmentation of OXT levels in an individual alters others’ perceptions and behavior toward an OXT-treated social partner. We examined social dynamics in well-established male-female pairs of marmoset monkeys (*Callithrix jacchus*) in which one member of the pair was administered an intranasal OXT agonist, an OXT antagonist (OXTA), or saline. OXT treatment did not alter the expression of affiliative toward an *untreated* partner. However, OXT did significantly influence the expression of proximity and grooming behavior with a *treated* partner, as a function of OXT treatment and sex. Female interest in initiating and maintaining proximity with a pair-mate was altered by OXT treatment. Untreated female marmosets departed from their saline-treated partner more frequently than they approached them, as indicated by a low proximity index score. However, when males received an intranasal OXT agonist they had a significantly increased proximity index score relative to saline, indicating that their *untreated* partner approached them more often than they departed from them). Saline-treated females initiated and received equivalent levels of grooming behavior. However, when female marmosets were treated with an OXT agonist their *untreated* partner groomed them proportionately more often, for a greater total duration, and for more time per bout, than they initiated grooming behavior. These results suggest that intranasal OXT altered male and female marmosets’ stimulus properties in such a way as to increase the amount of grooming behavior that females received from their long-term mate, as well as increase female interest in initiating and maintaining proximity with their long-term mate. Furthermore, these results support the notion that central OXT activity plays an important neuromodulatory role in the maintenance of long-lasting male-female relationships.

## Introduction

Adult male-female bonds function to facilitate reproduction, and lessen detrimental health outcomes due to stress and anxiety in socially monogamous species (Carter, 1998). Thus, forming and maintaining high-quality, long-lasting social relationships is critical for an individual’s survival and reproduction. Adult male-female bonds are partly characterized by initiating and maintaining close proximity with a social partner, as well as engaging in high levels of affiliative and sociosexual behavior (Kleiman, 1977). Despite extensive research on the neurobiological mechanisms fundamental to the formation of monogamous “pair-bonds” (Young et al., 2011), relatively little is known about the neuroendocrine underpinnings of maintaining that relationship (Resendez and Aragona, 2013).

There is considerable evidence that the oxytocin (OXT) system plays a critical role in the facilitation of social bonds (Lim and Young, 2006; Kendrick, 2000) and the expression of prosocial behavior toward a social partner, including affiliation (Feldman, 2012), cooperation (De Dreu, 2012), and trust (Mikolajczak et al., 2010), presumably by acting on OXT-relevant nuclei in the “social brain” (Macdonald and Macdonald, 2010). OXT is released endogenously following positive social interactions between existing social partners in humans (Grewen et al., 2005; Tops et al., 2007; Taylor et al., 2010) and nonhuman primates (Snowdon et al., 2010; Crockford et al., 2013). Additionally, pharmacological manipulation of the OXT system modulates social bonding behavior in socially monogamous mammals. Central administration of OXT reduces the duration required to form a stable pair bond (Williams et al., 1994), while administration of an OXT antagonist (OXTA) inhibits pair-bond formation in prairie voles (Williams et al., 1994). In newly-formed marmoset pairs, administration of an OXTA reduces measures of social proximity and food sharing (Smith et al., 2010). Thus, the OXT system has a critical role in the expression of partner-preference behavior and the formation of adult male-female bonds.

Though it is clear that the OXT system modulates the perceptions and behavior of an individual by acting on oxytocin receptors (OXTRs) in the “social brain”, less attention has been given to whether augmentation of OXT levels alters others’ perceptions and behavior *toward* an OXT-treated social partner. There is evidence in humans that maternal and paternal levels of plasma and salivary OXT are positively related to infant social engagement (Feldman et al., 2011). Furthermore, infants express more social behavior(s) in face-to-face interactions when their father receives intranasal OXT than when their father receives placebo, and experience a post-interaction increase in salivary OXT (Weisman et al., 2012). Moreover, intranasal OXT appears to influence several features of social cognition (e.g., face/emotion recognition, judgments of trustworthiness and attractiveness; Graustella and MacLeod, 2012; van IJzendoorn and Bakermans-Kranenburg, 2012). However, these studies on adults examined whether OXT influenced perceptions of other individuals’ attractiveness. In the current study we sought to examine if OXT treatment alters other’s behavior *toward* an OXT-treated individual, which may indicate that an OXT-treated individual is perceived as more socially attractive. To the extent that OXT facilitates social bonding, we might expect OXT to alter the stimulus properties of an individual as a way to enhance overall levels of affiliation and strengthen the social bond. This may occur by altering specific social cues that serve as a signal to direct more affiliative behavior toward an OXT-treated social partner. Thus, in the current study we examined the expression of social behavior of both an OXT-treated individual and their untreated long-term mate in marmoset monkeys.

Marmosets (*Callithrix jacchus*) are a highly social, cooperatively breeding New World primate that readily form and maintain long-term male-female relationships (Digby, 1995; Schaffner et al., 1995; Ågmo et al., 2012), and exhibit biparental and alloparental infant care (da Silva Mota et al., 2006). Like the socially monogamous prairie vole, marmosets employ a diverse set of socially monogamous mating strategies, including high levels of sociality between pair-mates (Evans, 1983; Schaffner et al., 1995) and aggressive responses to a potential rival (Ross et al., 2004). However, unlike prairie voles, which display a consistently strong partner preference, marmosets display a flexible pattern of sociosexual preferences (Smith et al., 2010; Cavanaugh et al., 2014), similar to those displayed by humans (Buss and Schmitt, 1993). For these reasons, the marmoset is an excellent translational primate model for the study of the maintenance of social bonds.

OXT amino-acid sequences were considered highly conserved among placental mammals (Acher, 1980; Caldwell and Young III, 2006; Donaldson and Young, 2008) until recently. Six distinct variants of OXT have been identified in New World monkeys (NWMs) and tree shrews (Lee et al., 2011; Ren et al., 2015; Vargas-Pinilla et al., 2015). Due to a proline substitution at the 8^th^ amino-acid position, the marmoset variant of OXT (Pro^8^-OXT) is distinctly different than the consensus mammalian variant of OXT (Leu^8^-OXT), and results in a substantially altered structure in the “linear” portion of the molecule that likely affects OXT ligand-OXTR binding, and subsequently behavior. Recently, we demonstrated that Pro^8^-OXT, but not Leu^8^-OXT, influences the expression of social behavior in well-established marmoset pairs during behavioral tasks when an opposite-sex stranger was present. Pro^8^-OXT facilitated fidelity in female marmosets by reducing time spent in close proximity with, and sociosexual behavior toward, an opposite-sex stranger (Cavanaugh et al., 2014), and reduced prosocial responses toward an opposite-sex stranger in male and female marmosets during an altruistic food-sharing task (Mustoe et al., 2015). Thus, species-specific OXT appears to decrease social motivation to interact with an opposite-sex stranger, thus reducing fidelity-threatening behaviors in well-established marmoset pairs. There is still relatively little known about the influence of the OXT system on the behavioral mechanisms that marmosets utilize to maintain their bond with a pair-mate. The expression of social behavior between pair-mates in their home-environment is particularly important; therefore, we examined how OXT agonists and an OXTA influenced affiliative and sociosexual behavior between pair-mates in well-established relationships.

In the current study, we sought to evaluate the role of OXT treatment on social interactions in well-established marmoset pairs. If the OXT system facilitates prosociality and bond maintenance, then marmosets treated with an OXT agonist should increase contact with, and display more affiliative behavior toward a long-term mate, while treatment with an OXTA should reduce the expression of prosocial behavior toward a long-term mate. Furthermore, if structural changes in the OXT ligand are biologically relevant, then marmosets treated with Pro^8^-OXT, but not Leu^8^-OXT, should increase contact with, and display more affiliative behavior toward a long-term mate. Moreover, if OXT influences an individual’s stimulus properties, then marmosets treated with an OXT agonist should display more social solicitation behavior, and receive more affiliative behavior from an untreated pair-mate.

## Materials and Methods

### Subjects

Six adult male and six adult female marmosets (*C. jacchus*), housed at the Callitrichid Research Center (CRC) at the University of Nebraska Omaha, were employed in this study. Animals were 3.2 ± 0.2 (mean ± SEM) years of age at the start of the study, and were kept in large indoor wire-mesh enclosures (1.0 × 2.5 × 2.0 m), equipped with a sleeping hammock, natural branches for climbing and various enrichment materials. Visual access was restricted between enclosures, but auditory and olfactory cues were not. Colony rooms at the CRC were maintained on a 12 h: 12 h light: dark cycle and at a temperature range between 19°C and 22°C. For all dietary and husbandry protocols please refer to Schaffner et al. (1995).

All males were surgically vasectomized prior to the study. All females received a 0.15 ml intra-muscular injection of cloprostenol (Estrumate^®^), a synthetic prostaglandin analog 3 days prior to each treatment period to synchronize females’ ovarian cycles by inducing luteolysis (Mustoe et al., 2012). The University of Nebraska Omaha/University of Nebraska Medical Center Institutional Animal Care and Use Committee evaluated and approved all procedures: 12–099–12-FC.

### Behavioral Paradigm

After eight weeks of cohabitation, which has been shown to be an adequate duration of time to allow for significant establishment of a male-female relationship in marmosets (Woodcock, 1982; Evans and Poole, 1984; Smith et al., 2011; Ågmo et al., 2012), subjects began treatment every other day across 7 days (Days 1, 3, 5, 7; this study occurred concurrently with Cavanaugh et al., 2014), in which data collection occurred on Day 9 of each treatment period). Marmosets were administered each treatment over four counterbalanced treatment periods. Furthermore, each member of the pair was treated individually during each treatment period in a counterbalanced order. There was an 11–13 day washout period between treatments.

Social interactions between pair-mates were observed 30 min post-treatment between 0900 and 1030 h for 20 min. Marmosets were habituated to the observers’ presence during the 4 month period leading up to the study, and were given 3–5 min to acclimate to the observer’s presence prior to behavioral recording. We measured the rate and duration of social interactions between pair-mates (i.e., affiliative, sociosexual, and aggressive/territorial behaviors; Table 1). All observers were trained to achieve a level of proficiency (κ > 0.90) on scoring affiliative, sociosexual, and aggressive/territorial behaviors. All focal animal observations were recorded using Stopwatch+ software (Emory University).

**Table 1:** **Ethogram of marmoset affiliative and social-proximity maintenance behavior patterns**.

Behavior	Operational definition
**Social behavior**
Solicit grooming^a^	Orientation of body or head to present to pair-mate for grooming
Grooming^a,b^	Manipulation of pelage of pair-mate by parting the fur with hands and removing particles with hands or teeth
Approach^a^	Moving to a distance of <10 cm from pair-mate
Leave^a^	Moving to a distance of >10 cm from pair-mate
Proximity^a,b^	Duration that marmoset spends within 10 cm of pair-mate
**Sexual behavior**
Open-mouth display^a^	A rhythmic opening and closing of the mouth (i.e., lip smacking, tongue flicking) directed toward a potential mate
Mount^a^	Male grasps female’s back and thrusts pelvis with an erect phallus
**Territorial behavior**
Scent mark^a^	Anogenital rubbing across a substrate, often preceded by gnawing on surface, or co-occurring with urination

*Note: Behaviors observed for all occurrences, ^a^frequency and ^b^duration*.

### Drug Treatments

#### Intranasal Administration of OXT Agonists

Marmosets were administered either saline or 25 IU (50μg/100μL saline solution) of one of two different OXT agonists, which yielded a dose of 150 μg/kg. The dose was determined based on previous primate literature (Heinrichs et al., 2003; Parker et al., 2005; Smith et al., 2010). Marmosets were administered Pro^8^-OXT (Anaspec Corp, California), Leu^8^-OXT (synthesized by Maurice Manning, University of Toledo), or saline 30 min prior to home-cage observations, via intranasal administration during a brief (~3 min) manual restraint. Intranasal administration was accomplished using a 100-μL Eppendorf pipette to administer 50-μL of solution to each nostril drop-wise (30 s between each nostril), and is a relatively well-tolerated, non-invasive method of administration.

Peptides administered intranasally are quickly absorbed into the bloodstream via the nasal passage, and some fraction of the peptides appear to bypass the blood-brain barrier (BBB) to access the central nervous system (CNS) via the olfactory bulb and the maxillary branch of the trigeminal nerve (Macdonald and Macdonald, 2010; Bethlehem et al., 2013). The neuropeptides OXT and arginine-vasopressin (AVP) are transported to the CNS and accumulate in the cerebrospinal fluid (CSF) in humans (Born et al., 2002; Striepens et al., 2013) and macaques (Chang et al., 2012; Dal Monte et al., 2014). In rats and mice, OXT levels were increased in microdialysates from the hippocampus and amygdala, and plasma, 30–60 min after intranasal administration (Neumann et al., 2013). Circulating levels of OXT persist for up to, but no more than 7 h in humans (van IJzendoorn et al., 2012). These results suggest that intranasal administration rapidly upregulates OXT levels in the brain and plasma during the timeframe of our behavioral testing, and that OXT clears the system several hours after testing.

#### Oral Administration of OXT Antagonist

Marmosets were treated with 20 mg/kg of an OXTA or saline 90 min prior to home-cage observations, via oral administration in a preferred food treat. The OXTA (L368,899^®^ provided by Dr. Peter Williams, Merck and Co., Inc.) is readily absorbed by the bloodstream after passage through the digestive system (Thompson et al., 1997), penetrates the CNS after peripheral administration, and accumulates in areas of the limbic system (Boccia et al., 2007).

### Data Analysis

To assess the possible effects of OXT treatment on patterns of sociality, data from home-cage observations were averaged across the 4 days of treatment for each treatment period to create a composite score. Raw values of proximity behavior (i.e., approach and depart from pair-mate), and grooming behavior (i.e., receive and initiate) were scored and analyzed for males and females in conditions when their pair-mate was treated and in conditions when they were treated. Several indices were also derived to assess whether males and females were proportionately more responsible for initiating and maintaining proximity with a treated partner, and whether treated males and females received proportionately more or less grooming from an untreated partner. The derived proximity index [i.e., (*Approach treated partner/Depart from treated partner*)] yielded scores from 0+ where a score > 1 indicates that marmosets approached their partner more frequently than they departed from them and a score < 1 indicates that marmosets departed from their partner more frequently than they approached them. The derived grooming indices [i.e., *(Receive groom/Total groom; Initiate groom/Total groom*)] yielded scores from –1.0 to +1.0, where +1.0 indicates that treated marmosets received all of the grooming behavior and –1.0 indicates that treated marmosets initiated in all of the grooming behavior. We used a similar calculation to create derived variables for total grooming duration and time spent grooming per bout. To account for within-pair interactions, these indices take into account the direction of the behavior of both the treated and untreated individual within the dyad. Consequently, the analysis of these indexes assesses the mean behavior of the interaction between two members of a dyad across OXT conditions. We are then able to extract the behavior of a particular individual by evaluating the change in the direction of the index variable relative to saline. The effect of sex and neuropeptide treatment (Pro^8^-OXT, Leu^8^-OXT, OXTA, saline) on social interactions with a pair-mate was evaluated using several mixed-model analyses of variance (ANOVAs). If main effects or interactions were significant, post-hoc comparisons were made using Fisher’s least significant difference. All alpha levels were set at *p* < 0.05.

## Results

**Figure 1:**
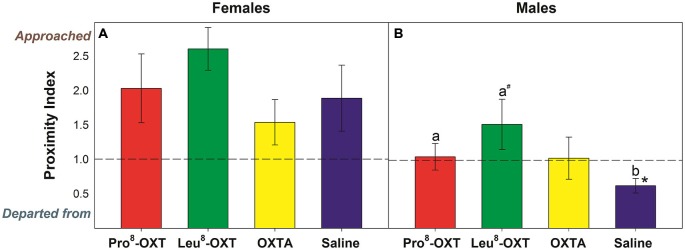
**Proximity index derived from the relative frequency that *untreated* (A) males and (B) females approached/departed from their *treated* partner.** A score > 1 indicates that marmosets approached their pair-mate more often than they departed from them, while a score < 1 indicates that marmosets departed from their pair-mate more often than they approached them. Data are expressed as a function of OXT treatment; a > b at *p* < 0.05 (^#^ indicates that a > b at *p* ≤ 0.10); Asterisks indicate significant difference from 1.0 (* < 0.05, ** < 0.01).

OXT treatment did not significantly influence how often a marmoset approached or departed from an *untreated* partner (*F*_(3,24)_ = 1.23, *p* > 0.05, η^2^ = 0.13); nor did OXT treatment interact with sex to modulate proximity behavior (*F*_(3,24)_ = 0.70, *p* > 0.05, η^2^ = 0.08). Yet, marmosets initiated and maintained proximity with their *treated* partner as a function of OXT treatment and sex (*F*_(3,27)_ = 3.25, *p* = 0.037, η^2^ = 0.27). Untreated female marmosets departed from their saline-treated male partner more frequently than they approached them (*t*_(5)_ = −3.59, *p* = 0.016), as indicated by a low proximity index score. However, males that received an OXT treatment had significantly increased proximity index scores, relative to saline (*F*_(3,12)_ = 3.66, *p* = 0.044, η^2^ = 0.48; Figure 1B). Proximity index scores were significantly increased in males that received Pro^8^-OXT (*t*_(5)_ = 3.86, *p* = 0.012), and moderately, but not significantly, increased in males that received Leu^8^-OXT (*t*_(5)_ = 2.02, *p* = 0.10), relative to saline. OXTA-treated males did not have significantly different proximity index scores than saline-treated males (*t*_(4)_ = 1.51, *p* = 0.21). Males did not alter the relative rate that they approached/departed from OXT-treated female partner, relative to their saline-treated female partner (*F*_(3,12)_ = 1.30, *p* > 0.05, η^2^ = 0.21; Figure 1A). See Table 2 for mean frequencies that males and females approached and departed from their partner, in conditions when their partner received a neuropeptide-treatment and in conditions when their partner was untreated.

**Table 2:** **Affiliative behavior (means and standard errors)**.

Behavior	Pro^8^-OXT	Leu^8^-OXT	OXTA	Saline	*F* value	*p* value
**Proximity behavior (treated female)**
Male approach female	7.13 (2.68)	8.88 (1.03)	7.75 (1.83)	9.63 (2.65)	0.40	0.75
Male depart from female	4.25 (1.84)	3.50 (0.32)	5.50 (1.43)	6.13 (2.13)	1.00	0.42
Female approach male	3.63 (1.00)	2.00 (0.60)	4.88 (1.41)	3.75 (0.93)	1.27	0.32
Female depart from male	6.25 (1.55)	7.13 (1.35)	6.50 (1.59)	6.50 (1.41)	0.60	0.98
**Proximity behavior (treated male)**
Female approach male	6.25 (1.19)	7.00 (1.56)	4.88 (1.74)	5.00 (1.57)	0.46	0.72
Female depart from male	6.75 (1.10)	5.88 (1.14)	4.63 (0.98)	8.75 (2.47)	1.39	0.28
Male approach female	6.63 (3.16)	8.25 (2.50)	4.63 (0.98)	6.89 (2.67)	0.66	0.59
Male depart from female	5.63 (1.92)	8.00 (2.82)	4.88 (1.24)	3.13 (1.15)	1.20	0.35
**Grooming behavior (treated female)**
Female receive	5.85 (2.14)	3.75 (1.26)	4.35 (1.45)	1.95 (1.58)	0.90	0.46
Female initiate	1.35 (0.90)	0.60 (0.44)	2.7 (1.02)	2.55 (0.70)	4.72	0.02
**Grooming behavior (treated male)**
Male receive	1.23 (0.72)	0.75 (0.75)	3.19 (2.71)	1.88 (0.72)	0.36	0.79
Male initiate	3.94 (1.80)	2.63 (0.72)	3.19 (1.28)	2.44 (0.99)	0.31	0.82

*Note: N = 12. The frequency (per hour of obs.) of each behavior is presented. Means (± SEM) are averaged across 4 days of treatment. Data are expressed as a function of OXT treatment: marmoset OXT variant (Pro^8^-OXT), consensus mammalian OXT variant (Leu^8^-OXT), OXT antagonist (OXTA: L368,899^®^), and saline*.

**Figure 2:**
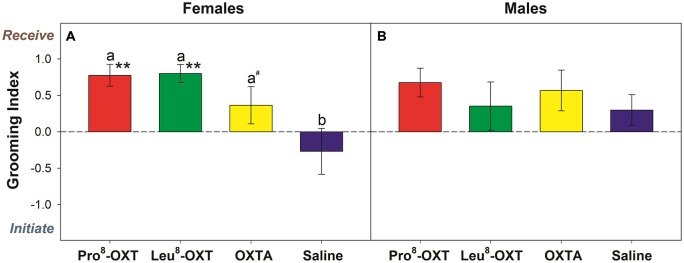
**Grooming index derived from the relative frequency that *treated* females (A) and males (B) received/initiated grooming behavior.** A positive score indicates that marmosets received grooming behavior more frequently than they initiated grooming behavior. A negative score indicates that marmosets initiated grooming behavior more often than they received grooming behavior. Data are expressed as a function of OXT treatment. a > b at *p* < 0.05 (^#^ indicates that a > b at *p* ≤ 0.10); Asterisks indicate significant difference from 0.0 (* < 0.05, ** < 0.01).

Marmosets also initiated and received grooming behavior differentially as a function of OXT treatment and sex (*F*_(3,24)_ = 6.13, *p* = 0.005, η^2^ = 0.50). Saline-treated females initiated and received equivalent levels of grooming behavior (*t*_(4)_ = 1.40, *p* = 0.23). However, when female marmosets received an OXT treatment they had a significantly increased grooming index score relative to saline (*F*_(3,12)_ = 9.12, *p* = 0.002, η^2^ = 0.70; Figure 2A). Female marmosets that received either intranasal Pro^8^-OXT (*t*_(4)_ = 5.17, *p* = 0.007) or Leu^8^-OXT (*t*_(5)_ = 7.91, *p* = 0.001) received grooming behavior proportionately more often than they initiated grooming behavior. Grooming index scores were significantly increased in females that received Pro^8^-OXT (*t*_(4)_ = 3.58, *p* = 0.023) or Leu^8^-OXT (*t*_(5)_ = 3.19, *p* = 0.024), and moderately, but not significantly, increased when females received an OXTA (*t*_(4)_ = 2.67, *p* = 0.06), relative to saline. Female marmosets that received an OXT treatment also had a significantly increased grooming index score for total duration (*F*_(3,12)_ = 5.37, *p* = 0.014, η^2^ = 0.57), and time per grooming bout (*F*_(3,12)_ = 4.92, *p* = 0.019, η^2^ = 0.55), relative to saline. Males that received an OXT treatment did not display a proportionate change in the relative frequency (*F*_(3,12)_ = 0.75, *p* > 0.05, η^2^ = 0.27; Figure 2B), total duration (*F*_(3,12)_ = 1.03, *p* > 0.05, η^2^ = 0.34), or time per bout (*F*_(3,12)_ = 1.72, *p* > 0.05, η^2^ = 0.46) that they received vs. initiated grooming behavior. Furthermore, males (*F*_(3,15)_ = 0.89, *p* > 0.05, η^2^ = 0.15) and females (*F*_(3,15)_ = 0.64, *p* > 0.05, η^2^ = 0.11) did not display changes in the frequency that they solicited grooming from their pair-mate when they were administered an OXT treatment. See Table 2 for mean frequencies that males and females initiated and received grooming from their partner, in conditions when their partner received a neuropeptide-treatment and in conditions when their partner was untreated.

Although males and females were differentially responsible for initiating and maintaining proximity with a pair-mate as a result of OXT treatment, overall proximity duration (*F*_(3,30)_ = 0.39, *p* > 0.05, η^2^ = 0.05) and time per proximity bout (*F*_(3,30)_ = 0.54, *p* > 0.05, η^2^ = 0.05) were unaffected by OXT treatment and sex of the individual. Overall levels of huddling duration (*F*_(3,30)_ = 0.54, *p* > 0.05, η^2^ = 0.05) and time per huddling bout (*F*_(3,30)_ = 0.82, *p* > 0.05, η^2^ = 0.08) were also unaffected by OXT treatment and sex of the individual. Male and female sexual-solicitation behavior (i.e., open-mouth display frequency), male mating behavior (i.e., mount frequency), and territorial behavior (i.e., scent mark frequency) did not vary as a function of OXT treatment (*p*’s > 0.05; Table 3).

**Table 3:** **Effects of OXT on sexual behavior and aggressive/territorial behavior**.

Behavior	Pro^8^-OXT	Leu^8^-OXT	OXTA	Saline	*F* value	*p* value
**Male mating behavior**
Mount	0.38 (0.20)	0.81 (0.44)	0.25 (0.13)	0.56 (0.22)	0.96	0.42
**Sexual solicitation behavior**
Open-mouth display	0.00 (0.00)	0.00 (0.00)	0.63 (0.06)	0.25 (0.25)	0.84	0.48
**Territorial behavior**
Scent mark	16.88 (2.73)	18.00 (3.32)	14.38 (1.94)	12.88 (2.20)	0.84	0.49

Note: N = 12. The frequency (per hour of obs.) of each behavior is presented. Means (±SEM) are averaged across 4 days of treatment. Data are expressed as a function of OXT treatment.

## Discussion

High-quality social interactions with a pair-mate are critical for the preservation of long lasting bonds between males and females in socially monogamous species. In the current study, modifying OXT activity via agonist and antagonist treatments significantly altered the stimulus properties of female marmosets in such a way as to increase the amount of grooming they received from their long-term mate. Furthermore, female marmosets displayed higher levels of proximity initiation and maintenance with an OXT-treated male partner, measured by a proportionate increase in how often females approached, relative to how often females departed from, their pair-mate. These results are taken in light of the finding that OXT did not alter obvious signs of social solicitation behavior. Thus, OXT appears to influence more subtle social signals that lead to an individual’s social partner increasing their expression of affiliative behavior. Furthermore, during social interactions between male and female marmosets, sexual and territorial/aggressive behaviors were unaffected by OXT manipulations, suggesting that the effects of OXT are exclusive to affiliative behavior in this social context.

The most conspicuous features of a high-quality sociosexual bond are the enhanced affiliative responses directed toward, and attachment with, a long-term mate. The sociosexual bond between male and female marmosets is partly reflected by the pervasive and reciprocal preference to engage in high levels of social and physical contact with a partner (Woodcock, 1982; Schaffner et al., 1995; Ågmo et al., 2012). Engaging in adequate levels of social and physical contact (e.g., initiating/maintaining proximity, grooming behavior) with a partner is essential for preserving an enduring sociosexual bond (Young, 2003). In the current study, female marmosets received proportionately more grooming behavior from their pair-mate than they expressed when they were treated with OXT. Yet, male marmosets did not receive more or less grooming behavior from their pair-mate than they expressed when they were treated with OXT. These results suggest that OXT may be altering certain unidentified stimulus properties of female, but not male, marmosets that increases females’ specific attractive qualities.

Interestingly, neither male nor female marmosets expressed more social solicitation behavior (i.e., solicit grooming) when treated with OXT, suggesting that their untreated partners may be responding to more subtle social signals. It is well known that marmosets have highly-attuned olfactory systems and utilize scent as a means of social communication (Epple, 1972; Smith et al., 2001). There is evidence from rodent and human literature that OXT action within the olfactory bulb enhances memory for a mother’s scent (Nagasawa et al., 2012). In the current study OXT may have increased the attractiveness of an individual’s scent, which led to increased interest in engaging in affiliative behavior with an OXT-treated individual. Alternatively, marmosets may have been reacting to an accumulation of social cues from multiples modes. Perhaps OXT-treated marmosets were making subtle changes in their body-position, body-motion, and/or vocalizations, and their highly attuned partner (untreated) was responsive to the accumulation of these changes via multisensory integration. The limited evidence on OXT and multisensory integration suggests that OXT may modulate parental head motion and proximity and infant-oriented gestures in humans (Weisman et al., 2012, 2013). Thus, marmosets may be responding to an accumulation of subtle signals from OXT-treated social partners.

Overall levels of grooming behavior were only moderately increased as a result of OXT treatment. However, the more noteworthy finding was that the rate an OXT-treated individual received grooming from their partner, relative to how often they initiated grooming behavior themselves, changed as a result of OXT treatment. Females treated with OXT initiated slightly less grooming and received slightly more grooming. Marmosets may maintain long-term bonds by expressing high-levels of affiliative behavior; they may be highly attuned to changes in the frequency and duration of grooming, as there may be an optimal level that is necessary to maintain a long-lasting bond. Thus, another possible explanation for OXT-related changes in affiliative behavior is that marmosets were responding to changes in the social dynamics of the relationships, and changed their behavior accordingly. Alternatively, OXT may increase the salience of their social partner, which may make them more trusting (Shamay-Tsoory et al., 2009) and receptive to the expression of affiliative behavior.

Engaging in physical and social contact with a long-term mate requires initiating and maintaining proximity. Female marmosets’ initiation and maintenance of proximity was altered when the OXT system was manipulated in their pair-mate. Under control conditions, males approached their pair-mate proportionately more often than they departed from them, suggesting that males were more responsible for initiating and maintaining proximity than females. Untreated female marmosets approached their OXT-treated male partner more and departed from them less. This suggests that treatment with OXT agonists may have the effect of increasing the “social attractiveness” of males, resulting in an increased number of approaches toward, and a decreased number of departures from. Interestingly, responsibility for initiating and maintaining proximity with a pair-mate was altered when males were treated with both the marmoset variant (Pro^8^), as well as the consensus-mammalian variant (Leu^8^) of OXT. The frequency that females initiated grooming, relative to received grooming, was also influenced by both OXT agonists. Thus, there is converging evidence that both the consensus-mammalian variant of OXT (Smith et al., 2010; Saito and Nakamura, 2011) as well as the marmoset variant of OXT (Cavanaugh et al., 2014; Mustoe et al., 2015) regulate affiliative and sociosexual behavior in marmosets.

Previous research has demonstrated ligand-specific modification of social behavior in well-established marmoset pairs during behavioral tasks when an opposite-sex stranger was present, with Pro^8^-OXT exerting more potent effects than Leu^8^-OXT (Cavanaugh et al., 2014; Mustoe et al., 2015). In the present study, it appears that both the Pro^8^ and Leu^8^ variants of OXT influenced social behavior between pair-mates. In a similar vein, intranasal administration of Leu^8^-OXT subtly augmented sociality in newly formed marmoset pairs (Smith et al., 2010), and intracerebroventricular (icv) administration of Leu^8^-OXT enhanced paternal tolerance for food sharing with offspring (Saito and Nakamura, 2011). The two OXT ligands have clear structural and physiochemical differences that likely produce differential binding affinity with the OXTR (Ren et al., 2015), and neural circuits underlying marmoset sociality may be differentially sensitive to the two forms of OXT. Furthermore, the threshold for alteration of social behavior by OXT ligand activation of the OXTR may differ across measures of social behavior (i.e., offspring care, food sharing, partner preference, proximity, grooming).

Intranasal administration of OXT, given in doses ranging from 24–48 IU, may enter the CNS via a transnasal route, but the majority of it appears to increase plasma OXT to supraphysiological levels (Leng and Ludwig, 2015). While it is predicted that intranasal OXT crosses the BBB via the olfactory bulb and the maxillary branch of the trigeminal nerve (Macdonald and Macdonald, 2010; Bethlehem et al., 2013), the exact transnasal-CNS route remains unknown, and the proportion of OXT that actually reaches the CNS is exceptionally small relative to the given dose (Born et al., 2002; Chang et al., 2012; Striepens et al., 2013; Dal Monte et al., 2014; Modi et al., 2014). Despite the small increases in CSF OXT levels after intranasal administration, intranasal OXT has profound and widespread effects on several features of social living (Bethlehem et al., 2013). One potential explanation is that intranasal OXT may not be acting directly on neural circuits that regulate social behavior, but instead may trigger endogenous OXT release via a peripheral feedback mechanism (Hurlemann and Scheele, 2015). Thus, treatment with either Pro^8^-OXT or Leu^8^-OXT in the current study may have been sufficient to trigger endogenous release by a peripheral feedback mechanism, subsequently altering the expression of social behavior.

In contrast to the strong effect of OXT agonists on affiliative behavior in well-established marmoset pairs, the OXTA had a limited effect, and only subtly altered marmoset sociality. Total proximity duration and time spent in proximity per bout, as well as total huddling duration and time spent huddling per bout were only marginally reduced when marmosets were treated with an OXTA, relative to saline, suggesting that both the overall duration and time per social interaction was diminished when endogenous OXT activity was suppressed. Interestingly, administration of an OXTA moderately, but not significantly, increased proximity index scores as well as grooming index scores relative to saline, in males and female respectively. One potential explanation of this unexpected finding is that administration of an OXTA altered some unmeasured aspect of behavior that subtly enhanced the individual’s receptiveness in such a way that their untreated partner was more motivated to approach them and groom them. Considering this modest effect, suppression of endogenous OXT activity does not seem to have a substantial effect on the expression of affiliative behavior in marmosets. The limited effect of OXTA may be due several possible reasons: (1) the acute nature of administration (i.e., every other day across 7 days); (2) the length of the relationship at the time of treatment; or (3) the pharmacological properties of the OXTA (L368,899^®^ which binds strongly to the OXTR, but is not selective for OXTR as it has affinity for V1aR as well; Manning et al., 2012). Previous research demonstrated that chronic OXTA administration reduced measures of food sharing, proximity, and huddling with a new pair-mate (Smith et al., 2010). Acute treatment of an OXTA in the current study may not have been sufficient to produce similar reductions in social behavior between pair-mates. Thus, well-established social relationships may be less sensitive to OXTA interventions, relative to the more dynamic social interactions during the establishment and development of new bonds.

Manipulation of the OXT-system differentially influenced affiliative behavior in male and female marmosets. Sex differences in neuropeptide-relevant nuclei in the “social brain” may explain the differential sensitivities, and the sexually dimorphic behavioral responses to OXT treatment. Similar to marmosets, central OXT activity influenced pair bonding behavior differentially in male and female prairie voles. Peripheral pulses of OXT in female, but not male, prairie voles facilitated the formation of a partner preference, while an OXTA blocked this effect in females (Cushing and Carter, 2000). In addition to the role of OXT in the modulation of social behavior between pair-mates, there is abundant evidence to suggest that AVP neural circuits are crucial to pair bonding in male prairie voles (Lim and Young, 2004; Young et al., 2011). Perhaps OXT and AVP receptors are differentially distributed in male and female marmosets, and this differential physiology contributes to the dissimilar behavioral patterns expressed during social interactions between pair-mates. While AVP immunereactive cells were found in several brain regions of the social behavior network (Wang et al., 1997a,b; Schorscher-Petcu et al., 2009), only the bed nucleus of the stria terminalis (BSNT) was found to be sexually dimorphic, with male marmosets expressing more AVP^+^ neurons than females (Wang et al., 1997a). However, male and female marmosets did not differ in the distribution of immunoreactive OXT neurons in the paraventricular and supraoptic nuclei of the hypothalamus, BNST, or medial amygdala (Wang et al., 1997a). More recently, there is increased interest in examining the distribution and characteristics of central OXT and AVP pathways in non-human primates (Freeman et al., 2014a,b). Further research in this area, particularly in the increasingly common biomedical animal model—the marmoset monkey, would greatly enhance our understanding of the neural mechanisms of action of these neuropeptides and their effects on social behavior.

Surprisingly, OXT treatment did not modulate sexual behavior between pair-mates. Marmosets administered an OXT agonist did not display more sexual solicitation behavior or copulatory behavior than when they were treated with saline. The OXT system has been implicated in female sexual receptivity (Cushing and Carter, 2000), as well as sexual arousal and orgasm (Behnia et al., 2014) during interactions between pair-mates. In a previous study, intranasal Pro^8^-OXT reduced the occurrence of sexual solicitation behavior toward an opposite-sex stranger (Cavanaugh et al., 2014), consequently reducing the likelihood of engaging in an extra-pair sexual encounter. Thus, the marmoset OXT system may modulate specific behavioral mechanisms that are context specific (i.e., reducing fidelity-threatening behaviors when an opposite-sex stranger is present; increasing affiliative behavior with a pair-mate in their home-environment); each critical for the preservation of a long-term relationship.

Adult sociosexual bonds in socially monogamous species function not only to facilitate reproduction, but to also reduce potentially deleterious health outcomes due to stress/anxiety (Carter, 1998). High-quality relationships and the associated social support from a long-term partner are associated with a host of health benefits, including improved disease outcomes, enhanced immune function, and reduced cardiovascular risk (Lillard and Waite, 1995; Kiecolt-Glaser and Newton, 2001). Therefore, behavioral strategies that help maintain long-term relationships are essential, and it appears that central OXT activity plays an important neuromodulatory role in the behavioral maintenance of social bonds. Affiliative contact with, and close proximity to, a long-term partner reduces the physiological and behavioral costs of stress and anxiety in marmosets (Smith et al., 1998). In the current study we demonstrated that administration of OXT agonists to a social partner increased the expression of affiliative behavior toward the OXT-treated individual. Thus, OXT treatment may be a means to increase social interest and expression of affiliative behavior in long-term social relationships. One potential limitation of the current study is non-independence between individuals within a social interaction, which is an unavoidable consequence of examining the complex interactions of dyadic relationships. We have approached the dynamic social interactions that comprise a dyadic relationship from a fresh perspective, by exploring the behavior of both individuals simultaneously, as well as examining if one member of the pair altered their expression of affiliative behavior when their pair-mate received an OXT treatment.

The results of this study suggest that intranasal OXT administration altered male and female stimulus properties in such a way as to increase the amount of grooming behavior that females received from their untreated social partner, as well as increase female interest in initiating and maintaining proximity with an OXT-treated partner. Although we did not identify changes in specific social solicitation behaviors in male and females that received intranasal OXT, it is clear that marmosets expressed higher levels of affiliative behavior toward their pair-mate when their pair-mate had augmented OXT levels, compared to control conditions. The results of the present study further support the notion that central OXT activity plays a role in the maintenance of well-established male-female relationships in adults. Furthermore, they suggest that intranasal OXT may enhance the initiation of social interaction through proximity behavior, as well as enhancing the “social attractiveness” of an OXT-treated individual during a social interaction. Future work will attempt to quantify specific, and certainly subtle, changes in the behavior of OXT-treated individuals that may render them more attractive as social partners.

## Author Contributions

JC designed the study, wrote the protocol, collected the data, conducted the statistical analysis, and wrote the first draft of the manuscript. MH and AH assisted in data collection and provided comments/edits on previous drafts of the manuscript. JF provided comments/edits on previous drafts of the manuscript. All authors contributed to and have approved the final manuscript.

## Funding

This work was supported in part by funds from the National Institutes of Health (HD 042882) awarded to Jeffrey A. French, and by funds from University of Nebraska Omaha’s Graduate Research and Creative Activity Committee awarded to Jon Cavanaugh.

## Conflict of Interest Statement

The authors declare that the research was conducted in the absence of any commercial or financial relationships that could be construed as a potential conflict of interest.
